# Antimicrobial resistance and genomic rep-PCR fingerprints of *Pseudomonas aeruginosa* strains from animals on the background of the global population structure

**DOI:** 10.1186/s12917-017-0977-8

**Published:** 2017-02-21

**Authors:** Isa Serrano, Manuela Oliveira, José Pedro Santos, Florence Bilocq, Alexandre Leitão, Luis Tavares, Jean-Paul Pirnay, Daniel De Vos

**Affiliations:** 1 CIISA/Faculty of Veterinary Medicine, University of Lisbon, Lisbon, Portugal; 2 Laboratory for Molecular and Cellular Technology, Queen Astrid Military Hospital, Brussels, Belgium

**Keywords:** Animal origin, Antimicrobials, Environment, MDR, Non-clonal, Population, Pseudomonas aeruginosa, Rep-PCR, Serotype, XDR

## Abstract

**Background:**

*Pseudomonas aeruginosa* is an important human opportunistic pathogen responsible for fatal nosocomial infections worldwide, and has emerged as a relevant animal pathogen. Treatment options are dramatically decreasing, due to antimicrobial resistance and the microorganism’s large versatile genome.

Antimicrobial resistance profiles, serotype frequency and genomic profile of unrelated *P. aeruginosa* isolates of veterinary origin (*n* = 73), including domesticated, farm, zoo and wild animals mainly from Portugal were studied. The genomic profile, determined by DiversiLab system (Rep-PCR-based technique), was compared with the *P. aeruginosa* global population structure to evaluate their relatedness.

**Results:**

Around 40% of the isolates expressed serotypes O6 (20.5%) and O1 (17.8%). A total of 46.6% of isolates was susceptible to all antimicrobials tested. Isolates obtained from most animals were non-multidrug resistant (86.3%), whereas 11% were multidrug resistant, MDR (non-susceptible to at least one agent in ≥ three antimicrobial categories), and 2.7% extensively drug resistant, XDR (non-susceptible to at least one agent in all but two or fewer antimicrobial categories). Resistance percentages were as follows: amikacin (0.0%), aztreonam (41.1%), cefepime (9.6%), ceftazidime (2.7%), ciprofloxacin (15.1%), colistin (0.0%), gentamicin (12.3%), imipenem (1.4%), meropenem (1.4%), piperacillin + tazobactam (12.3%), ticarcillin (16.4%), ticarcillin + clavulanic acid (17.8%), and tobramycin (1.4%).

Animal isolates form a population with a non-clonal epidemic structure indistinguishable from the global *P. aeruginosa* population structure, where no specific ‘animal clonal lineage’ was detected.

**Conclusions:**

Serotypes O6 and O1 were the most frequent. Serotype frequency and antimicrobial resistance patterns found in *P. aeruginosa* from animals were as expected for this species. This study confirms earlier results that *P. aeruginosa* has a non-clonal population structure, and shows that *P. aeruginosa* population from animals is homogeneously scattered and indistinguishable from the global population structure.

**Electronic supplementary material:**

The online version of this article (doi:10.1186/s12917-017-0977-8) contains supplementary material, which is available to authorized users.

## Background

*Pseudomonas aeruginosa* is an infectious bacterial species, with a world-wide dispersion, capable to infect and promote disease in different tissues [1], and responsible for remarkable morbidity and mortality rates in humans [2].

*P. aeruginosa* is a ubiquitous bacterial species, with an exceptional capacity to adapt to different aquatic and terrestrial ecological niches [3, 4]. Its high adaptability is in part explainable by its metabolic versatility [4–6]. This opportunistic bacterium migrates from its natural environment, colonizing and infecting a wide range of organisms [5, 6], including plants [7] and different animals, such as fruit flies [8], waxmoth [9], zebrafish [10] and mammals [11, 12].

In veterinary medicine, *P. aeruginosa* is responsible for a large variety of infections such as ocular infections [13], mastitis [14, 15], discospondylitis [16], otitis [17, 18], pyoderma [19], ulcerative keratitis [20], urinary tract infections [21] and incision, joint, invasive device and wound infections [22]. The most imminent problems are otitis externa in dogs, mastitis in cattle, dermatitis in rabbits and fleece rot in sheep. However, the importance of animal infections caused by *P. aeruginosa* has been disregarded due to the low detection of animal clinical cases [23].

Overall, infections caused by *P. aeruginosa* are hard to treat because this bacterium shows intrinsic and acquired resistance to different antimicrobial compounds [6]. It has been argued that the presence of specific outer membrane proteins involved in efflux transport systems or affecting cell permeability may explain the intrinsic resistance of *P. aeruginosa* to antimicrobials [24–26]. *P. aeruginosa* strains are also able to express a wide variety of acquired resistance mechanisms, including the production of β-lactamases and carbapenemases [14], mainly due to mutations in chromosomal resistance genes or acquisition of resistance genes from other bacteria through plasmids [18]. Moreover, resistance transmission from humans to animals has been acknowledged [27, 28].

Different studies have been consensual about the fact that *P. aeruginosa* clinical isolates are indistinguishable from the ones from the environment, either genotypically, chemotaxonomically, or functionally [5, 29, 30]. In order to understand the global population structure, clinical and environmental isolates collected around the world were recently analyzed [4, 5]. Authors concluded that *P. aeruginosa* has an epidemic population structure, which is non-clonal. There are no specific *P. aeruginosa* clones related with specific diseases, habitat or animal species [5, 23].

The objective of this study was to characterize the antimicrobial resistance patterns, serotype frequency, and genomic profile of a collection of *P. aeruginosa* isolates from a wide variety of animal species with different kinds of infections, from different geographic regions of Portugal. A rapid and accurate genotyping method based on Rep-PCR, the DiversiLab system, was used and the genomic profile was then compared with the *P. aeruginosa* global population structure to evaluate their relatedness.

## Methods

### *P. aeruginosa* isolates

In this study, a total of 73 *P. aeruginosa* isolates were used, belonging to a collection of animal isolates from the Faculty of Veterinary Medicine, Technical University of Lisbon (Additional file 1: Table S1). Isolates were collected from 2003–2012 in different geographic regions of Portugal, except for one sea turtle isolate sampled in Principe Island in the Gulf of Guinea. Our batch included 50 isolates from pets [dog (*n* = 39), cat (*n* = 5), parrot (*n* = 4), turtle (*n* = 2)], 15 from farm animals [horse (*n* = 6), cow (*n* = 5), sheep (*n* = 2), pig (*n* = 1), goat (*n* = 1)], 7 from zoo animals [seal (*n* = 1), dolphin (*n* = 2), kangaroo (*n* = 2), tamarind (*n* = 2)] and one from a wild free-living sea turtle.

Bacteria were isolated on Columbia blood agar (COS, BioMérieux, Marcy-l’Etoile, France), identified through their morphologic characteristics and biochemical profile (API 20 NE, BioMérieux, Marcy-l’Etoile, France) and kept frozen at -80 °C until further processing. *P. aeruginosa* control strain ATCC 27853 was used in this study.

### Molecular identification

Species identification of the isolates was confirmed using a previously described PCR assay targeting the *P. aeruginosa* species-specific *oprI* and *oprL* genes [24].

### Serotyping

Isolates were grown overnight on Luria-Bertani agar medium (Gibco-BRL-Life Technologies, Brussels, Belgium) at 37 °C, after which were serotyped by slide agglutination according to the International Antigenic Typing Scheme (IATS) for *P. aeruginosa* [31], using a panel of 16 type O monovalent antisera (Bio-Rad, Temse, Belgium). Four antisera pools, each containing four antisera, were also used: pool A (O1, O3, O4, O6); pool E (O2, O5, O15, O16); pool C (O9, O10, O13, O14); and pool F (O7, O8, O11, O12).

### Antimicrobial resistance

Antimicrobial resistance studies were performed by using the Vitek 2 system (BioMerieux, Marcy-l’Etoile, France) in accordance with the manufacturer’s instructions. *P. aeruginosa* ATCC 27853 was included as control strain. The following antimicrobials were tested: amikacin, aztreonam, cefepime, ceftazidime, ciprofloxacin, colistin, gentamicin, imipenem, meropenem, piperacillin + tazobactam, ticarcillin, ticarcillin + clavulanic acid, and tobramycin. Antimicrobial resistance phenotypes, represented by the minimum inhibitory concentrations (MICs) were interpreted according to CLSI guidelines: CLSI, Performance Standards for Antimicrobial Disk and Dilution Susceptibility Tests for Bacteria Isolated From Animals [32]. The breakpoints of aztreonam, cefepime, ceftazidime, ciprofloxacin, colistin, meropenem, piperacillin + tazobactam, and tobramycin were not defined at the above CLSI, so their MICs were interpreted using: CLSI Performance Standards for Antimicrobial Susceptibility Testing [33].

### Rep-PCR genotyping

DiversiLab system (rep-PCR) was used for isolates genotyping. *P. aeruginosa* DNA was extracted using the UltraCleanTM Microbial DNA Isolation Kit (Mo Bio Laboratories Inc., Solana Beach, CA) according to the manufacturer’s instructions. DNA quantification was measured using a NanoDrop ND-1000 spectrophotometer (NanoDrop Technologies, Wilmington, DE, USA). Then, rep-PCR was performed using PTC 200 thermocycler and *Pseudomonas* fingerprinting kit (Bacterial Barcodes, bioMe´rieux, Athens, GA, USA) in a total reaction volume of 25 μl. The reaction mixture consisted of 18 μl of rep-PCR MM1, 2.5 μl of Gene Amp 10X, 2 μl of primer Mix, 0.5 μl of AmpliTaq DNA polymerase (Applied Biosystems, Foster City, CA, USA) and 2 μl of genomic DNA (25–50 ng/ml). Thermal conditions were as follows: initial denaturation step at 94 °C for 2 min, 35 cycles including denaturation at 94 °C for 30 s, annealing at 50 °C for 30 s and extension at 70 °C for 90 s, followed by a final extension step at 70 °C for 3 min.

PCR products were analyzed using the Agilent 2100 BioAnalyzer (Agilent Technologies, Santa Clara, CA, USA). Then, the amplified fragments (sizes from 100–1000 bp) were electrophoretically separated using a microfiluidic labchip. In order to monitor reproducibility, the *P. aeruginosa* ATCC 27853 reference strain was used as a control in each PCR reaction and in each chip. Electropherograms were downloaded and automatically analyzed by the DiversiLab software (version 3.4) (BioMerieux, Brussels, Belgium). All fingerprint patterns were normalized, then, the Pearson correlation coefficient was used in order to calculate the distance matrices among all samples. Based on UPGMA and multidimensional scaling, the DiversiLab software created a customized report presenting a dendrogram, electropherograms, virtual gel images and scatter plots. Relatedness among isolates was deduced as previously described [34]: isolates showing similarity levels above 95% were considered as linked, while isolates with similarity levels below 95% were considered as different. Closely related isolates differing by a maximum of two band classes were collapsed into one node.

## Results

### Molecular identification and serotype distribution

Both target genes used in rep-PCR (*oprI* and *oprL*) were detected in all 73 *P. aeruginosa* isolates tested, confirming their identification as *Pseudomonas aeruginosa*. Isolates were serotyped, being observed that most isolates belonged to serotypes O6 (20.5%), O1 (17.8%), O11 (9.6%), O3 (5.5%) and O9 (5.5%) (Table 1). Isolates belonging to serotypes O4, O10, O15 and O12 were also detected, but at a lower percentage. PA isolates (strains that polyagglutinate in pools E (O2 + O5 + O15 + O16) and F (O7, O8, O11, O12), but not in the individual antisera) were 12.3%. Nine isolates (12.3%) were classified as NT/NA: NT (Non Typeable strains which are polyagglutinating), NA (strains that do Not Agglutinate in any pool or individual antiserum) (Table 1).

**Table 1:** Serotype percentage of *P. aeruginosa* animal isolates (*n* = 73)

Serotype	Number	Percentage
**O6**	**15**	**20.5**
**O1**	**13**	**17.8**
**PA**	**9**	**12.3**
**NT/NA**	**9**	**12.3**
**O11**	**7**	**9.6**
**O3**	**4**	**5.5**
**O9**	**4**	**5.5**
O4	3	4.1
O10	3	4.1
O15	3	4.1
O12	2	2.7

Serotypes with a percentage of more than 5% are expressed in boldface

NT, Non Typeable strains which are polyagglutinating; PA, strains that PolyAgglutinate in pools E (O2 + O5 + O15 + O16) and F (O7, O8, O11, O12), but not in the individual antisera; NA, strains that do Not Agglutinate in any pool or individual antiserum

### Antimicrobial resistance

A total of 46,6% of isolates was susceptible to all antimicrobials tested. Table 2 summarizes the frequency of resistant isolates for all antimicrobials tested, where intermediate isolates are also classified as resistant (low-level resistance). Resistance to aztreonam (41.1%) was the highest. Resistance to ciprofloxacin (15.1%), piperacillin + tazobactam (12.3%), ticarcillin (16.4%) and ticarcillin + clavulanic acid (17.2%), was between 10% and 20%. Resistance was lowest for cefepime (9.6%), ceftazidime (2.7%), imipenem (1.4%), gentamicin (12.3%), meropenem (1.4%), and tobramycin (1.4%). All isolates were susceptible to amikacin and to colistin (Table 2).

**Table 2:** Antimicrobial resistance of *P. aeruginosa* isolates (*n* = 73)

		Resistance % (n)		
Antimicrobials	MIC breakpoint (μg/mL)	High^a^	Low^b^	Total
Amikacin	≤16 - ≥64	0.0	0.0	0.0
Aztreonam	≤8 - ≥32	15.1 (11)	26.0 (19)	41.1 (30)
Cefepime	≤8 - ≥32	0.0	9.6 (7)	9.6 (7)
Ceftazidime	≤8 - ≥32	0.0	2.7 (2)	2.7 (2)
Ciprofloxacin	≤1 - ≥4	15.1 (11)	0.0	15.1 (11)
Colistin	≤2 - ≥8	0.0	0.0	0.0
Gentamicin	≤4 - ≥16	2.7 (2)	9.6 (7)	12.3 (9)
Imipenem	≤4 - ≥16	1.4 (1)	0.0	1.4 (1)
Meropenem	≤2 - ≥8	1.4 (1)	0.0	1.4 (1)
Piperacillin + tazobactam	≤16/4 - ≥128/4	1.4 (1)	11.0 (8)	12.3 (9)
Ticarcillin	≤64 - ≥128	16.4 (12)	0.0	16.4 (12)
Ticarcillin + clavulanic acid	≤64/2 - ≥128/2	17.8 (13)	0.0	17.8 (13)
Tobramycin	≤4 - ≥16	1.4 (1)	0.0	1.4 (1)

^a^High, high-level resistant; ^b^Low, low-level resistant (Intermediate)

The number of isolates with high-level resistance is higher than the number of isolates with low-level resistance (intermediate) (Table 2).

The distribution of antimicrobial resistance per serotype was as follows: serotype O1 isolates were resistant to aztreonam (46.2%), cefepime (7.7%), ciprofloxacin (7.7%), gentamicin (7.7%), piperacillin + tazobactam (15.4%), ticarcillin (15.4%), and ticarcillin + clavulanic acid (15.4%); serotype O6 isolates were resistant to aztreonam (26.7%), ciprofloxacin (20.0%), gentamicin (6.7%), ticarcillin (13.3%), and ticarcillin + clavulanic acid (13.3%). These two serotypes, representing 38% of animal isolates, are in accordance to the total results, except the fact that serotype O6 was fully susceptible to the two cephalosporins tested, whereas the total resistance for cephalosporins was 2.7% (ceftazidime) and 9.6% (cefepime).

Multidrug resistant (MDR) isolates are non-susceptible to at least one agent in ≥ three antimicrobial categories, and extensively drug resistant (XDR) isolates non-susceptible to at least one agent in all but two or fewer antimicrobial categories [35]. According to Magiorakos AP et al., 2012 [33], ticarcillin is not used to define MDR and XDR isolates, thus it was not used to define MDR and XDR isolates in this study. Isolates obtained from most animals were non-multidrug resistant (nor MDR, nor XDR) (86.3%). Only 2.7% of the isolates were XDR and 11.0% were MDR.

MDR was found in isolates belonging to serotypes O1, O6, O11, O15, and to PA and NT isolates. MDR profiles observed were as follows: aztreonam / ciprofloxacin / ticarcillin + clavulanic acid (3/8) aztreonam / cefepime / ticarcillin + clavulanic acid / piperacillin + tazobactam (1/8), aztreonam / ceftazidime / ticarcillin + clavulanic acid / piperacillin + tazobactam (1/8), aztreonam / ciprofloxacin / gentamicin / ticarcillin + clavulanic acid (1/8), aztreonam / cefepime / gentamicin / ticarcillin + clavulanic acid / piperacillin + tazobactam (1/8), aztreonam / cefepime / ciprofloxacin / ticarcillin + clavulanic acid / piperacillin + tazobactam (1/8).

The two XDR isolates were obtained only from dogs. One isolate expressed serotype O12 and was susceptible to only one antimicrobial category (polymyxin) and non-susceptible to at least one agent in all other antimicrobial categories (including aminoglycosides, as it was non-susceptible to gentamicin). The other XDR isolate, was a PA isolate fully susceptible to two antimicrobial categories (carbapenems and polymyxin) and non-susceptible to at least one agent in all other antimicrobial categories (including cephalosporins, as it was non-susceptible to cefepime, and aminoglycosides, as it was non-susceptible to gentamicin).

### Genotyping

Fully automated rep-PCR (DiversiLab system) was used as a rapid method to genetically analyze the population structure of *P. aeruginosa*. The total genome profile was determined by rep-PCR and combined with former data from clinical (animal and human) and environmental *P. aeruginosa* isolates collected across the world, previously studied through a polyphasic approach consisting of three outer membrane (lipo)protein gene sequences (*oprI*, *oprL* and *oprD*), amplified fragment length polymorphism, serotype and pyoverdine type [4].

**Fig. 1:**
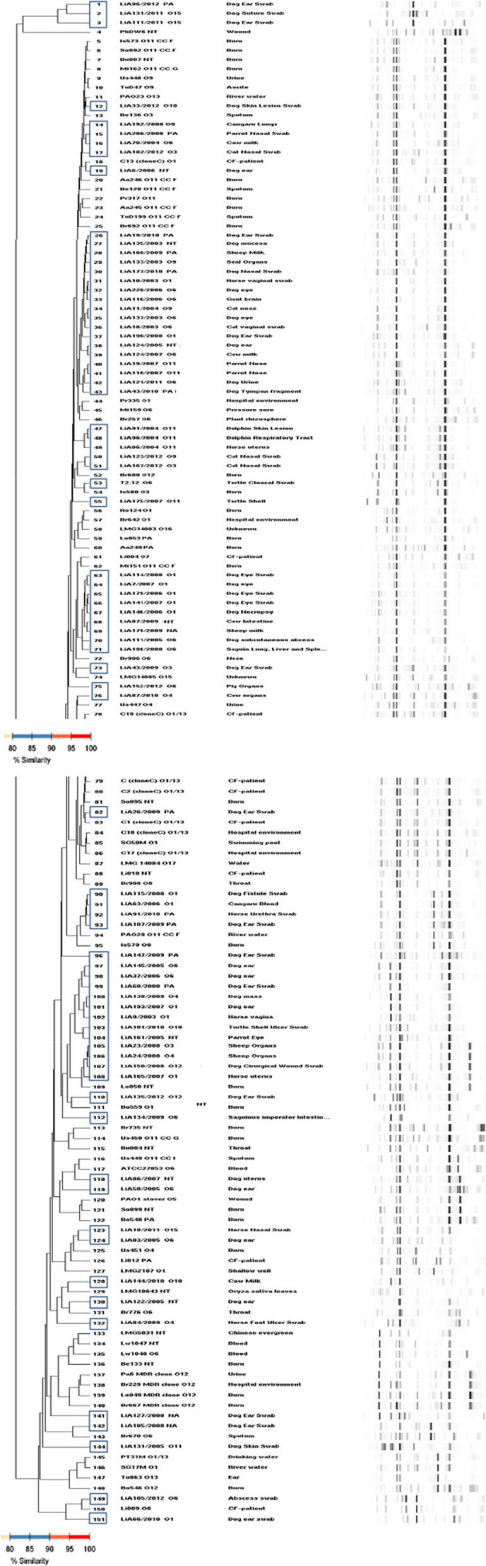
Normalized rep-PCR patterns and dendrogram for a total of 151 *P. aeruginosa* isolates: 73 *P. aeruginosa* isolates of this study plus 5 animal isolates not fully characterized (all delineated in blue), and 73 *P. aeruginosa* isolates from clinic (animal and human) and environmental origin from a previous study [4]. DiversiLab dendrogram based on UPGMA and Pearson correlation coefficient. Percentages of similarity are shown below the dendrogram

Genotyping with rep-PCR did not show any animal specific cluster, as showed in Fig 1. The dendrogram included a total of 151 *P. aeruginosa* isolates: 73 from this study, and 5 isolates which were not fully characterized from animals collected in Portugal (these 78 are delineated in blue), and 73 *P. aeruginosa* isolates from clinic (animal and human) and environmental samples from a previous study [4]. Considering a similarity of ≥80% clusters obtained included isolates from all origins: animal, human and environmental (Fig. 1). In fact, all animal isolates from this study, including the 78 animal clinical isolates collected from 2003–2012 in Portugal and the one sea turtle isolate sampled in the Gulf of Guinea, were homogeneously scattered in the global *P. aeruginosa* population structure previously depicted by Pirnay et al., (2002) [4].

## Discussion

Rep-PCR is a rapid method for bacterial genome fingerprinting, based on highly conserved repetitive sequence elements [repetitive extragenic palindromic (REP) sequence] within bacterial genomes, which are amplified by PCR, allowing the analyses of strain-specific patterns [36]. DiversiLab system used in our study is more reproducible than standard rep-PCR method, as the analysis is fully automated [37].

Multilocus sequence typing (MLST) is a strain-typing system that focuses exclusively on seven conserved housekeeping genes. The analysis of these genes, less likely to undergo horizontal gene transfer because of selective neutrality, provides a more accurate imprint of the recombination effect [5, 38]. The combination of alleles at the seven loci provided an allelic profile, or sequence type (ST). The relatedness between isolates is determined through a dendrogram from the matrix of pairwise differences between STs [39]. These data can be used in different aspects of evolutionary biology, whether to understand the evolution of genetic lineages or to estimate the relative contribution of recombination and single mutations in bacterial species [40]. MLST is therefore suitable for phylogenetic analysis enabling to track the global clonal history of the species with high accuracy [38]. Another advantage of MLST is that sequence data are portable between laboratories enabling to compare data from different geographic areas [40].

Although MLST is highly informative, the genetic variation indexed by MLST accumulates slowly, being more useful for long term epidemiological studies [39]. It has a limited resolution when applied to closely related stains [37]. On the other hand, DiversiLab is efficient to detect subtle genomic differences when applied to very closely related strains, being more useful for short term studies and outbreaks investigation [37].

Although we did not perform MLST, DiversiLab system results allow concluding that all animal isolates from this study were homogeneously scattered in the global *P. aeruginosa* population structure (Fig. 1), confirming earlier results that *P. aeruginosa* has a non-clonal population structure [4].

Our results are in accordance with the previous study by Pirnay et al., (2002) [4], in which a total of 73 *P. aeruginosa* clinical and environmental isolates collected worldwide presented predominantly the following serotypes: O1 (12.3%), O6 (10.9%), O11 (15.1%). The only exception was for serotype O12, which was less frequent in our study (2.7% versus 9.6%) (Table 1) [4]. Our results also agree with a recent study [41], regarding *P. aeruginosa* isolates from nosocomial pneumonia cases from several hospitals located in different countries. In both studies, frequency of serotypes O6, O11, O1, O4, O12 and O15 was similar, as also observed for not typeable isolates. By the contrary, different results were observed regarding serotype O10 (4.1% versus 9.8%), and we were not able to detect serotypes O2, O7 and O8 (Table 1).

*P. aeruginosa* is generally resistant against a wide spectrum of antimicrobial agents. Hence, only a few agents, including some extended-spectrum β-lactams, aminoglycosides, and fluoroquinolones are still effective [42, 43]. Resistance patterns were typical for this species. Near 50% of isolates was susceptible to all antimicrobials tested (46.6%). As expected, the aminoglycosides, carbapenems, cephalosporins, and polymyxin studied had also low resistance levels, maximum 12.3% (Table 2), thus amikacin, cefepime, ceftazidime, colistin, gentamicin, imipenem, meropenem, and tobramycin are generally effective in animals. Of notice that carbapenems are critically important antimicrobials and should be reserved for human use. The latest European Medicines Agency report, published in October 2015, shows that sales of antimicrobials for use in animals in Europe fell by approximately 8% between 2011 and 2013. However, there are notably differences between countries: 11 countries have decreased the sales of veterinary antimicrobial agents, whereas 6 countries including Portugal increased [44]. The greater percentages of sales for food-producing animals, including horses, in mg per population correction unit (mg/PCU), of the various veterinary antimicrobial classes, in Portugal, in 2013, were tetracyclines (39.2), penicillins (16.9), macrolides (12.4), polymyxins (10.1) [44].

There was a high resistance rate among animal isolates tested against monobactam aztreonam (41.1%). Resistance rates of penicillins [ticarcillin (16.4%), ticarcillin + clavulanic acid (17.8%), piperacillin + tazobactam (12.3%)] and the fluoroquinolone ciprofloxacin (15.1%) were inferior to 20% (Table 2).

The higher resistance rates among those critical antimicrobials should most probably result from intrinsic antimicrobial resistance traits of *P. aeruginosa*, such as low cell wall permeability, and its capacity to express acquired resistance mechanisms [14], and to a lesser degree to veterinary medicine antimicrobial consumption. It should be addressed that probably this collection of strains from animals are probably more resistant than in general, since often specimens are collected from chronic cases.

Near 14% of isolates were MDR and XDR (13.7%), pointing to the importance of the transmission of antimicrobial resistance genes in *P. aeruginosa* from animals. The antimicrobial resistance is a priority to FAO/OIE/WHO under the concept “One Health”. Is crucial to include an international perspective in any politics about antimicrobial resistance. To decrease antimicrobial resistance in veterinary isolates, it is important to continue to implement responsible-use campaigns, to raise awareness of the threat of antimicrobial resistance, and to restrict its use and targets [44]. In the future, a safe and gradual implementation of alternatives to antimicrobials will be crucial to face antimicrobial resistance [45].

Interestingly, the XDR isolate only susceptible to amikacin, colistin, and tobramycin, was isolated from a dog with an infected surgical wound and was serotyped as O12 which is a serotype associated with human XDR clonal-cluster [5, 46]. Based on this observation, can one can speculate the possibility of resistance transmission from humans to animals [27, 28].

## Conclusions

The reported data shows that serotype frequency and the resistance patterns of *P. aeruginosa* collected from animals were typical for this species. The intrinsic resistance mechanism of *P. aeruginosa* should explain the presence of multidrug profiles and the high resistance rates observed in some critical antimicrobials. This study confirms earlier results that *P. aeruginosa* has a non-clonal population structure, and demonstrates the similarity between environmental and clinical isolates from animals and humans. They are all part of the global *P. aeruginosa* population and form a non-clonal epidemic structure. No specific ‘animal clonal lineage’ could be detected.

## Additional file

Additional file 1: Table S1. Overview of the characteristics, serotyping and antimicrobial susceptibility of *P. aeruginosa* strains. (DOCX 30 kb)

## References

[1] Lyczak JB, Cannon CL, Pier GB (2000). Establishment of Pseudomonas aeruginosa infection: lessons from a versatile opportunist. Microbes Infect.

[2] Talbot GH, Bradley J, Edwards JE, Gilbert D, Scheld M, Bartlett JG (2006). Bad bugs need drugs: an update on the development pipeline from the Antimicrobial Availability Task Force of the Infectious Diseases Society of America. Clin Infect Dis.

[3] Goldberg JB (2000). Pseudomonas: global bacteria. Trends Microbiol.

[4] Pirnay JP, De Vos D, Cochez C, Bilocq F, Vanderkelen A, Zizi M, Ghysels B, Cornelis P (2002). Pseudomonas aeruginosa displays an epidemic population structure. Environ Microbiol.

[5] Pirnay JP, Bilocq F, Pot B, Cornelis P, Zizi M, Van Eldere J, Deschaght P, Vaneechoutte M, Jennes S, Pitt T (2009). Pseudomonas aeruginosa population structure revisited. PloS One.

[6] Silby MW, Winstanley C, Godfrey SA, Levy SB, Jackson RW (2011). Pseudomonas genomes: diverse and adaptable. FEMS Microbiol Rev.

[7] Rahme LG, Ausubel FM, Cao H, Drenkard E, Goumnerov BC, Lau GW, Mahajan-Miklos S, Plotnikova J, Tan MW, Tsongalis J (2000). Plants and animals share functionally common bacterial virulence factors. Proc Natl Acad Sci USA.

[8] Apidianakis Y, Rahme LG (2009). Drosophila melanogaster as a model host for studying Pseudomonas aeruginosa infection. Nat Protoc.

[9] Miyata S, Casey M, Frank DW, Ausubel FM, Drenkard E (2003). Use of the Galleria mellonella caterpillar as a model host to study the role of the type III secretion system in Pseudomonas aeruginosa pathogenesis. Infect Immun.

[10] Clatworthy AE, Lee JS, Leibman M, Kostun Z, Davidson AJ, Hung DT (2009). Pseudomonas aeruginosa infection of zebrafish involves both host and pathogen determinants. Infect Immun.

[11] Potvin E, Lehoux DE, Kukavica-Ibrulj I, Richard KL, Sanschagrin F, Lau GW, Levesque RC (2003). In vivo functional genomics of Pseudomonas aeruginosa for high-throughput screening of new virulence factors and antibacterial targets. Environ Microbiol.

[12] Snouwaert JN, Brigman KK, Latour AM, Malouf NN, Boucher RC, Smithies O, Koller BH (1992). An animal model for cystic fibrosis made by gene targeting. Science.

[13] Ledbetter EC, Mun JJ, Kowbel D, Fleiszig SM (2009). Pathogenic phenotype and genotype of Pseudomonas aeruginosa isolates from spontaneous canine ocular infections. Invest Ophthalmol Vis Sci.

[14] Nam HM, Lim SK, Kang HM, Kim JM, Moon JS, Jang KC, Kim JM, Joo YS, Jung SC (2009). Prevalence and antimicrobial susceptibility of gram-negative bacteria isolated from bovine mastitis between 2003 and 2008 in Korea. J Dairy Sci.

[15] Sela S, Hammer-Muntz O, Krifucks O, Pinto R, Weisblit L, Leitner G (2007). Phenotypic and genotypic characterization of Pseudomonas aeruginosa strains isolated from mastitis outbreaks in dairy herds. J Dairy Res.

[16] Sura R, Creden A, Van Kruiningen HJ (2008). Pseudomonas-associated discospondylitis in a two-month-old llama. J Vet Diagn Invest.

[17] McKay L, Rose CD, Matousek JL, Schmeitzel LS, Gibson NM, Gaskin JM (2007). Antimicrobial testing of selected fluoroquinolones against Pseudomonas aeruginosa isolated from canine otitis. J Am Anim Hosp Assoc.

[18] Rubin J, Walker RD, Blickenstaff K, Bodeis-Jones S, Zhao S (2008). Antimicrobial resistance and genetic characterization of fluoroquinolone resistance of Pseudomonas aeruginosa isolated from canine infections. Vet Microbiol.

[19] Hillier A, Alcorn JR, Cole LK, Kowalski JJ (2006). Pyoderma caused by Pseudomonas aeruginosa infection in dogs: 20 cases. Vet Dermatol.

[20] Lin CT, Petersen-Jones SM (2008). Antibiotic susceptibility of bacteria isolated from cats with ulcerative keratitis in Taiwan. J Small Anim Pract.

[21] Werckenthin C, Alesik E, Grobbel M, Lubke-Becker A, Schwarz S, Wieler LH, Wallmann J (2007). Antimicrobial susceptibility of Pseudomonas aeruginosa from dogs and cats as well as Arcanobacterium pyogenes from cattle and swine as determined in the BfT-GermVet monitoring program 2004-2006. Berl Munch Tierarztl Wochenschr.

[22] Weese JS (2008). A review of multidrug resistant surgical site infections. Vet Comp Orthop Traumatol.

[23] Haenni M, Hocquet D, Ponsin C, Cholley P, Guyeux C, Madec JY, Bertrand X (2015). Population structure and antimicrobial susceptibility of Pseudomonas aeruginosa from animal infections in France. BMC Vet Res.

[24] De Vos D, Lim A, Pirnay JP, Struelens M, Vandenvelde C, Duinslaeger L, Vanderkelen A, Cornelis P (1997). Direct detection and identification of Pseudomonas aeruginosa in clinical samples such as skin biopsy specimens and expectorations by multiplex PCR based on two outer membrane lipoprotein genes, oprI and oprL. J Clin Microbiol.

[25] Masuda N, Sakagawa E, Ohya S (1995). Outer membrane proteins responsible for multiple drug resistance in Pseudomonas aeruginosa. Antimicrob Agents Chemother.

[26] Nikaido H (1994). Prevention of drug access to bacterial targets: permeability barriers and active efflux. Science.

[27] Mohan K, Fothergill JL, Storrar J, Ledson MJ, Winstanley C, Walshaw MJ (2008). Transmission of Pseudomonas aeruginosa epidemic strain from a patient with cystic fibrosis to a pet cat. Thorax.

[28] Wang Y, Wang X, Schwarz S, Zhang R, Lei L, Liu X, Lin D, Shen J (2014). IMP-45-producing multidrug-resistant Pseudomonas aeruginosa of canine origin. J Antimicrob Chemother.

[29] Alonso A, Rojo F, Martinez JL (1999). Environmental and clinical isolates of Pseudomonas aeruginosa show pathogenic and biodegradative properties irrespective of their origin. Environ Microbiol.

[30] Romling U, Wingender J, Muller H, Tummler B (1994). A major Pseudomonas aeruginosa clone common to patients and aquatic habitats. Appl Environ Microbiol.

[31] Liu PV, Wang S (1990). Three new major somatic antigens of Pseudomonas aeruginosa. J Clin Microbiol.

[32] CLSI (2015). Performance Standards for Antimicrobial Disk and Dilution Susceptibility Tests for Bacteria Isolated from Animals.

[33] CLSI (2015). Performance Standards for Antimicrobial Susceptibility Testing.

[34] Doleans-Jordheim A, Cournoyer B, Bergeron E, Croize J, Salord H, Andre J, Mazoyer MA, Renaud FN, Freney J (2009). Reliability of Pseudomonas aeruginosa semi-automated rep-PCR genotyping in various epidemiological situations. Eur J Clin Microbiol Infect Dis.

[35] Magiorakos AP, Srinivasan A, Carey RB, Carmeli Y, Falagas ME, Giske CG, Harbarth S, Hindler JF, Kahlmeter G, Olsson-Liljequist B (2012). Multidrug-resistant, extensively drug-resistant and pandrug-resistant bacteria: an international expert proposal for interim standard definitions for acquired resistance. Clin Microbiol Infect.

[36] Olive DM, Bean P (1999). Principles and applications of methods for DNA-based typing of microbial organisms. J Clin Microbiol.

[37] Maatallah M, Bakhrouf A, Habeeb MA, Turlej-Rogacka A, Iversen A, Pourcel C, Sioud O, Giske CG (2013). Four genotyping schemes for phylogenetic analysis of Pseudomonas aeruginosa: comparison of their congruence with multi-locus sequence typing. PLoS One.

[38] Khan NH, Ahsan M, Yoshizawa S, Hosoya S, Yokota A, Kogure K (2008). Multilocus sequence typing and phylogenetic analyses of Pseudomonas aeruginosa Isolates from the ocean. Appl Environ Microbiol.

[39] Enright MC, Spratt BG (1998). A multilocus sequence typing scheme for Streptococcus pneumoniae: identification of clones associated with serious invasive disease. Microbiology.

[40] Spratt BG (1999). Multilocus sequence typing: molecular typing of bacterial pathogens in an era of rapid DNA sequencing and the internet. Curr Opin Microbiol.

[41] Lu Q, Eggimann P, Luyt CE, Wolff M, Tamm M, Francois B, Mercier E, Garbino J, Laterre PF, Koch H (2014). Pseudomonas aeruginosa serotypes in nosocomial pneumonia: prevalence and clinical outcomes. Crit Care (London, England).

[42] Harada K, Arima S, Niina A, Kataoka Y, Takahashi T (2012). Characterization of Pseudomonas aeruginosa isolates from dogs and cats in Japan: current status of antimicrobial resistance and prevailing resistance mechanisms. Microbiol Immunol.

[43] Lister PD, Wolter DJ, Hanson ND (2009). Antibacterial-resistant Pseudomonas aeruginosa: clinical impact and complex regulation of chromosomally encoded resistance mechanisms. Clin Microbiol Rev.

[44] EMA - European Medicines Agency. Sales of veterinary antimicrobial agents in 26 EU/EEA countries in 2013 (EMA/387934/2015). In: European Surveillance of Veterinary Antimicrobial Consumption; 2015.

[45] Oliveira M, Serrano I (eds.). Frontiers in antimicrobial agents - The challenges of antimicrobial resistance in the development of new therapeutics. Sharjah: Bentham Science Publishers; 2016.

[46] Pirnay JP, De Vos D, Cochez C, Bilocq F, Pirson J, Struelens M, Duinslaeger L, Cornelis P, Zizi M, Vanderkelen A (2003). Molecular epidemiology of Pseudomonas aeruginosa colonization in a burn unit: persistence of a multidrug-resistant clone and a silver sulfadiazine-resistant clone. J Clin Microbiol.

