# Supplementary material for: Antimicrobial resistance and genomic rep-PCR fingerprints of Pseudomonas aeruginosa strains from animals on the background of the global population structure
Source: BMC Vet Res. 2017 Feb 21;13:58. doi: 10.1186/s12917-017-0977-8 (PMC5319083; doi:10.1186/s12917-017-0977-8)
Supplement: Additional file 1: Table S1. — Overview of the characteristics, serotyping and antimicrobial susceptibility of P. aeruginosa strains. (DOCX 30 kb) [file 12917_2017_977_MOESM1_ESM.docx]

**Table S1. Overview of the characteristics, serotyping and antimicrobial susceptibility of *P. aeruginosa* strains.**

|  | | | | | **Penicillin** | | | **Caphalosporin** | | **Monobactam** | **Carbapenem** | | **Aminoglycoside** | | | **Fluoroquinolone** | **Polymyxin** |
| --- | --- | --- | --- | --- | --- | --- | --- | --- | --- | --- | --- | --- | --- | --- | --- | --- | --- |
| **Isolate** | **Species** | **Source** | **Year** | **Serotype** | **TIC** | **TCC** | **TZP** | **CAZ** | **FEP** | **ATM** | **IPM** | **MEM** | **AN** | **GM** | **TM** | **CIP** | **CS** |
| LiA135/2003 | Pet | Dog mucosa | 2003 | NT | 32 / S | 16 / S | 8 / S | 2 / S | 2 / S | 4 / S | 2 / S | 1 / S | ≤ 2 / S | ≤ 1 / S | ≤ 1 / S | ≤ 0,25 / S | 1 / S |
| LiA137/2003 | Pet | Dog eye | 2003 | 6 | 32 / S | 32 / S | 8 / S | 4 / S | 2 / S | 4 / S | 2 / S | ≤ 0,25 / S | ≤ 2 / S | ≤ 1 / S | ≤ 1 / S | ≤ 0,25 / S | 2 / S |
| LiA18/2003 | Pet | Cat vagina | 2003 | 6 | 32 / S | 16 / S | 8 / S | 2 / S | ≤ 1 / S | 4 / S | 1 / S | ≤ 0,25 / S | ≤ 2 / S | ≤ 1 / S | ≤ 1 / S | ≤ 0,25 / S | ≤ 0,5 / S |
| LiA11/2004 | Pet | Cat nose | 2004 | 9 | 32 / S | 32 / S | 8 / S | 2 / S | ≤ 1 / S | 16 / I | 1 / S | 0,5 / S | ≤ 2 / S | ≤ 1 / S | ≤ 1 / S | ≤ 0,25 / S | ≤ 0,5 / S |
| LiA111/2005 | Pet | Dog subcutaneous abcess | 2005 | 6 | 64 / S | 64 / S | 16 / S | 4 / S | 2 / S | 16 / I | 2 / S | ≤ 0,25 / S | ≤ 2 / S | ≤ 1 / S | ≤ 1 / S | ≤ 0,25 / S | 1 / S |
| LiA122/2005 | Pet | Dog ear | 2005 | NT | 32 / S | 32 / S | 8 / S | 2 / S | 4 / S | 4 / S | 2 / S | 1 / S | 4 / S | 4 / S | ≤ 1 / S | ≤ 0,25 / S | ≤ 0,5 / S |
| LiA124/2005 | Pet | Dog ear | 2005 | NT | ≥ 128 / R | ≥ 128 / R | 16 / S | 2 / S | 2 / S | 16 / I | 1 / S | 0,5 / S | ≤ 2 / S | ≤ 1 / S | ≤ 1 / S | ≥ 4 / R | ≤ 0,5 / S |
| LiA131/2005 | Pet | Dog skin | 2005 | 11 | 32 / S | 32 / S | 8 / S | 2 / S | 2 / S | 4 / S | 1 / S | ≤ 0,25 / S | ≤ 2 / S | ≤ 1 / S | ≤ 1 / S | ≤ 0,25 / S | 1 / S |
| LiA145/2005 | Pet | Dog ear | 2005 | 6 | 16/ S | 16/ S | ≤ 4 / S | 2 / S | 4 / S | 2 / S | 2 / S | 0,5 / S | 4 / S | 8 / I | ≤ 1 / S | ≤ 0,25 / S | ≤ 0,5 / S |
| LiA161/2005 | Pet | Parrot | 2005 | NT | ≥ 128 / R | ≥ 128 / R | 64 / I | 16 /I | 8 / S | 64 / R | 1 / S | 1 / S | ≤ 2 / S | ≤ 1 / S | ≤ 1 / S | 1 / S | 2 / S |
| LiA50/2005 | Pet | Dog ear | 2005 | 6 | ≤ 8 / S | 16/ S | ≤ 4 / S | ≤ 1 / S | ≤ 1 / S | 2 / S | 1 / S | ≤ 0,25 / S | ≤ 2 / S | ≤ 1 / S | ≤ 1 / S | ≤ 0,25 / S | 2 / S |
| LiA83/2005 | Pet | Dog ear | 2005 | 6 | 32 / S | 32 / S | 8 / S | 4 / S | 2 / S | 4 / S | 2 / S | ≤ 0,25 / S | ≤ 2 / S | ≤ 1 / S | ≤ 1 / S | ≤ 0,25 / S | ≤ 0,5 / S |
| LiA146/2006 | Pet | Dog pleural fluid | 2006 | 1 | 32 / S | 16/ S | ≤ 4 / S | 2 / S | ≤ 1 / S | 4 / S | 1 / S | 0,5 / S | ≤ 2 / S | ≤ 1 / S | ≤ 1 / S | ≤ 0,25 / S | ≤ 0,5 / S |
| LiA179/2006 | Pet | Dog eye | 2006 | 1 | ≥ 128 / R | ≥ 128 / R | 32 / I | 4 / S | 4 / S | 32 / R | 1 / S | 1 / S | ≤ 2 / S | ≤ 1 / S | ≤ 1 / S | ≤ 0,25 / S | ≤ 0,5 / S |
| LiA228/2006 | Pet | Dog eye | 2006 | 6 | 32 / S | 16 / S | ≤ 4 / S | 4 / S | 2 / S | 8 / S | 2 / S | ≤ 0,25 / S | 4 / S | 2 / S | ≤ 1 / S | ≤ 0,25 / S | 1 / S |
| LiA37/2006 | Pet | Dog ear | 2006 | 6 | 16 / S | 16 / S | 8 / S | 2 / S | 8 / S | 4 / S | 1 / S | ≤ 0,25 / S | ≤ 2 / S | 2 / S | ≤ 1 / S | ≥ 4 / R | ≤ 0,5 / S |
| **Isolate** | **Species** | **Source** | **Year** | **Serotype** | **TIC** | **TCC** | **TZP** | **CAZ** | **FEP** | **ATM** | **IPM** | **MEM** | **AN** | **GM** | **TM** | **CIP** | **CS** |
| LiA6/2006 | Pet | Dog ear | 2006 | NT | 32 / S | 16 / S | 8 / S | 4 / S | 8 / S | 4 / S | 2 / S | 0,5 / S | 16 / S | 16 / R | 2 / S | ≤ 0,25 / S | ≤ 0,5 / S |
| LiA118/2007 | Pet | Parrot | 2007 | 11 | ≥ 128 / R | ≥ 128 / R | 32 / I | 4 / S | 16 / I | ≥ 64 / R | 1 / S | 1 / S | 8 / S | 8 / I | ≤ 1 / S | 1 / S | ≤ 0,5 / S |
| LiA141/2007 | Pet | Dog eye | 2007 | 1 | ≤ 8 / S | 16/ S | ≤ 4 / S | ≤ 1 / S | 2 / S | 2 / S | 1 / S | ≤ 0,25 / S | ≤ 2 / S | 2 / S | ≤ 1 / S | ≤ 0,25 / S | ≤ 0,5 / S |
| LiA175/2007 | Pet | Turtle | 2007 | 11 | 32 / S | 16 / S | 8 / S | 2 / S | 2 / S | 4 / S | 1 / S | ≤ 0,25 / S | ≤ 2 / S | ≥ 16 / R | ≥ 16 / R | ≥ 4 / R | ≤ 0,5 / S |
| LiA19/2007 | Pet | Parrot | 2007 | 11 | 32 / S | 16 / S | 8 / S | 2 / S | 4 / S | 4 / S | 1 / S | ≤ 0,25 / S | 8 / S | 8 / I | ≤ 1 / S | ≤ 0,25 / S | ≤ 0,5 / S |
| LiA193/2007 | Pet | Dog ear | 2007 | 1 | 64 / S | 64 / S | 8 / S | 4 / S | 2 / S | 16 / I | 2 / S | ≤ 0,25 / S | 4 / S | 2 / S | ≤ 1 / S | ≤ 0,25 / S | ≤ 0,5 / S |
| LiA7/2007 | Pet | Dog eye | 2007 | 1 | 32 / S | 16 / S | 8 / S | 2 / S | ≤ 1 / S | 4 / S | 1 / S | ≤ 0,25 / S | ≤ 2 / S | ≤ 1 / S | ≤ 1 / S | ≤ 0,25 / S | ≤ 0,5 / S |
| LiA86/2007 | Pet | Dog uterus | 2007 | NT | 64 / S | ≥ 128 / R | 32 / I | 4 / S | 2 / S | 16 / I | 2 / S | 1 / S | ≤ 2 / S | ≤ 1 / S | ≤ 1 / S | ≤ 0,25 / S | ≤ 0,5 / S |
| LiA105/2008 | Pet | Dog | 2008 | NA | 32 / S | 32 / S | 8 / S | 4 / S | 2 / S | 16 / I | ≤ 0,25 / S | ≤ 0,25 / S | 8 / S | 2 / S | ≤ 1 / S | ≤ 0,25 / S | ≤ 0,5 / S |
| LiA114/2008 | Pet | Dog | 2008 | 1 | ≤ 8 / S | 16 / S | ≤ 4 / S | ≤ 1 / S | 2 / S | 2 / S | 1 / S | ≤ 0,25 / S | 8 / S | 8 / I | ≤ 1 / S | ≤ 0,25 / S | ≤ 0,5 / S |
| LiA115/2008 | Pet | Dog | 2008 | 1 | ≥ 128 / R | ≥ 128 / R | 32 / I | 8 / S | 16 / I | ≥ 64 / R | 2 / S | 1 / S | 4 / S | 4 / S | ≤ 1 / S | 1 / S | ≤ 0,5 / S |
| LiA127/2008 | Pet | Dog | 2008 | NA | 16 / S | 16 / S | ≤ 4 / S | ≤ 1 / S | ≤ 1 / S | 2 / S | 1 / S | ≤ 0,25 / S | ≤ 2 / S | ≤ 1 / S | ≤ 1 / S | ≤ 0,25 / S | ≤ 0,5 / S |
| LiA150/2008 | Pet | Dog | 2008 | 12 | ≥ 128 / R | ≥ 128 / R | ≥ 128 / R | 16 / I | 16 / I | ≥ 64 / R | ≥ 16 / R | ≥ 16 / R | 8 / S | 8 / I | ≤ 1 / S | ≥ 4 / R | ≤ 0,5 / S |
| LiA190/2008 | Pet | Dog | 2008 | 1 | 32 / S | 32 / S | 8 / S | 2 / S | 2 / S | 16 / I | 1 / S | ≤ 0,25 / S | ≤ 2 / S | ≤ 1 / S | ≤ 1 / S | ≥ 4 / R | ≤ 0,5 / S |
| LiA206/2008 | Pet | Parrot | 2008 | PA | 32 / S | 32 / S | 8 / S | 2 / S | 2 / S | 2 / S | 2 / S | ≤ 0,25 / S | ≤ 2 / S | ≤ 1 / S | ≤ 1 / S | ≤ 0,25 / S | ≤ 0,5 / S |
| LiA60/2008 | Pet | Dog | 2008 | PA | 32 / S | 32 / S | 8 / S | 2 / S | ≤ 1 / S | 16 / I | 1 / S | ≤ 0,25 / S | ≤ 2 / S | ≤ 1 / S | ≤ 1 / S | 1 / S | ≤ 0,5 / S |
| LiA107/2009 | Pet | Dog | 2009 | PA | ≥ 128 / R | ≥ 128 / R | 32 / I | 8 / S | 16 / I | ≥ 64 / R | ≤ 0,25 / S | 1 / S | ≤ 2 / S | 2 / S | ≤ 1 / S | ≥ 4 / R | ≤ 0,5 / S |
| LiA138/2009 | Pet | Dog | 2009 | 4 | 32 / S | 16 / S | ≤ 4 / S | 2 / S | ≤ 1 / S | 4 / S | 2 / S | 1 / S | ≤ 2 / S | ≤ 1 / S | ≤ 1 / S | ≤ 0,25 / S | ≤ 0,5 / S |
| LiA147/2009 | Pet | Dog | 2009 | PA | 32 / S | 32 / S | 8 / S | 4 / S | 2 / S | 8 / S | 2 / S | 1 / S | ≤ 2 / S | ≤ 1 / S | ≤ 1 / S | ≤ 0,25 / S | 1 / S |
| LiA26/2009 | Pet | Dog | 2009 | PA | 64 / S | 64 / S | 8 / S | 4 / S | 2 / S | 16 / I | 2 / S | ≤ 0,25 / S | ≤ 2 / S | ≤ 1 / S | ≤ 1 / S | ≤ 0,25 / S | ≤ 0,5 / S |
| LiA43/2009 | Pet | Dog | 2009 | 3 | ≤ 8 / S | ≤ 8 / S | 8 / S | 2 / S | 8 / S | ≤ 1 / S | ≤ 0,25 / S | ≤ 0,25 / S | ≤ 2 / S | ≤ 1 / S | ≤ 1 / S | ≥ 4 / R | ≤ 0,5 / S |
| **Isolate** | **Species** | **Source** | **Year** | **Serotype** | **TIC** | **TCC** | **TZP** | **CAZ** | **FEP** | **ATM** | **IPM** | **MEM** | **AN** | **GM** | **TM** | **CIP** | **CS** |
| LiA101/2010 | Pet | Turtle | 2010 | 10 | 32 / S | 32 / S | 8 / S | 4 / S | 2 / S | 16 / I | 1 / S | 0,5 / S | ≤ 2 / S | ≤ 1 / S | ≤ 1 / S | ≤ 0,25 / S | ≤ 0,5 / S |
| LiA177/2010 | Pet | Dog | 2010 | PA | ≤ 8 / S | ≤ 8 / S | ≤ 4 / S | ≤ 1 / S | ≤ 1 / S | ≤ 1 / S | 1 / S | ≤ 0,25 / S | ≤ 2 / S | ≤ 1 / S | ≤ 1 / S | ≤ 0,25 / S | ≤ 0,5 / S |
| LiA43/2010 | Pet | Dog | 2010 | PA | ≥ 128 / R | ≥ 128 / R | 32 / I | 4 / S | 16 / I | ≥ 64 / R | 2 / S | 2 / S | 8 / S | 8 / I | ≤ 1 / S | ≥ 4 / R | ≤ 0,5 / S |
| LiA66/2010 | Pet | Dog | 2010 | 1 | 32 / S | 32 / S | 8 / S | 2 / S | 2 / S | 4 / S | 1 / S | ≤ 0,25 / S | ≤ 2 / S | ≤ 1 / S | ≤ 1 / S | ≤ 0,25 / S | ≤ 0,5 / S |
| LiA111/2011 | Pet | Dog | 2011 | 15 | 32 / S | 16 / S | 8 / S | 2 / S | 8 / S | 4 / S | ≤ 0,25 / S | ≤ 0,25 / S | 4 / S | 4 / S | ≤ 1 / S | 1 / S | ≤ 0,5 / S |
| LiA121/2011 | Pet | Dog | 2011 | 6 | ≥ 128 / R | ≥ 128 / R | 16 / S | 4 / S | 8 / S | 32 / R | 2 / S | 2 / S | 4 / S | 4 / S | ≤ 1 / S | ≥ 4 / R | ≤ 0,5 / S |
| LiA131/2011 | Pet | Dog | 2011 | 15 | ≥ 128 / R | ≥ 128 / R | 8 / S | 4 / S | 8 / S | 16 / I | 2 / S | 4 / S | 4 / S | 8 / I | ≤ 1 / S | ≥ 4 / R | ≤ 0,5 / S |
| LiA123/2012 | Pet | Cat | 2012 | 9 | ≤ 8 / S | ≤ 8 / S | 8 / S | 4 / S | 16 / I | 2 / S | 2 / S | ≤ 0,25 / S | ≤ 2 / S | 4 / S | ≤ 1 / S | ≤ 0,25 / S | ≤ 0,5 / S |
| LiA135/2012 | Pet | Dog | 2012 | 12 | 32 / S | 16 / S | 8 / S | 4 / S | 2 / S | 4 / S | ≤ 0,25 / S | ≤ 0,25 / S | ≤ 2 / S | ≤ 1 / S | ≤ 1 / S | ≤ 0,25 / S | ≤ 0,5 / S |
| LiA167/2012 | Pet | Cat | 2012 | 3 | ≤ 8 / S | ≤ 8 / S | ≤ 4 / S | 2 / S | 16 / I | ≤ 1 / S | 2 / S | ≤ 0,25 / S | 4 / S | 4 / S | ≤ 1 / S | ≤ 0,25 / S | ≤ 0,5 / S |
| LiA182/2012 | Pet | Cat | 2012 | 3 | 64 / S | 64 / S | 8 / S | 4 / S | 2 / S | 16 / I | 2 / S | ≤ 0,25 / S | ≤ 2 / S | ≤ 1 / S | ≤ 1 / S | ≤ 0,25 / S | ≤ 0,5 / S |
| LiA33/2012 | Pet | Dog | 2012 | 10 | ≥ 128 / R | ≥ 128 / R | 16 / S | 4 / S | 8 / S | ≥ 64 / R | 1 / S | 1 / S | ≤ 2 / S | ≤ 1 / S | ≤ 1 / S | 1 / S | ≤ 0,5 / S |
| LiA96/2012 | Pet | Dog | 2012 | PA | 16 / S | 16 / S | 8 / S | ≤ 1 / S | ≤ 1 / S | 2 / S | 2 / S | ≤ 0,25 / S | ≤ 2 / S | ≤ 1 / S | ≤ 1 / S | ≤ 0,25 / S | ≤ 0,5 / S |
| LiA19/2003 | Farm | Horse vagina | 2003 | 1 | 32 / S | 64 / S | 8 / S | 4 / S | 2 / S | 16 / I | 1 / S | ≤ 0,25 / S | ≤ 2 / S | ≤ 1 / S | ≤ 1 / S | ≤ 0,25 / S | ≤ 0,5 / S |
| LiA9/2003 | Farm | Horse vagina | 2003 | 1 | 32 / S | 32 / S | ≤ 4 / S | 2 / S | ≤ 1 / S | 4 / S | 1 / S | ≤ 0,25 / S | ≤ 2 / S | ≤ 1 / S | ≤ 1 / S | ≤ 0,25 / S | ≤ 0,5 / S |
| LiA70/2004 | Farm | Cow milk | 2004 | 6 | 32 / S | 32 / S | 8 / S | 2 / S | ≤ 1 / S | 4 / S | 1 / S | 0,5 / S | ≤ 2 / S | ≤ 1 / S | ≤ 1 / S | ≤ 0,25 / S | ≤ 0,5 / S |
| LiA86/2004 | Farm | Horse uterus | 2004 | 11 | 32 / S | 32 / S | 8 / S | 4 / S | 2 / S | 16 / I | 2 / S | 0,5 / S | ≤ 2 / S | ≤ 1 / S | ≤ 1 / S | 1 / S | ≤ 0,5 / S |
| LiA116/2006 | Farm | Goat brain | 2006 | 6 | 32 / S | 16/ S | 8 / S | 2 / S | 2 / S | 4 / S | 2 / S | ≤ 0,25 / S | ≤ 2 / S | ≤ 1 / S | ≤ 1 / S | ≤ 0,25 / S | ≤ 0,5 / S |
| LiA124/2007 | Farm | Cow milk | 2007 | 6 | ≥ 128 / R | ≥ 128 / R | 16 / S | 2 / S | 2 / S | 16 / I | 1 / S | 0,5 / S | ≤ 2 / S | ≤ 1 / S | ≤ 1 / S | ≥ 4 / R | ≤ 0,5 / S |
| LiA165/2007 | Farm | Horse uterus | 2007 | 1 | 32 / S | 32 / S | 8 / S | 4 / S | 2 / S | 4 / S | 1 / S | ≤ 0,25 / S | ≤ 2 / S | ≤ 1 / S | ≤ 1 / S | ≤ 0,25 / S | ≤ 0,5 / S |
| LiA23/2008 | Farm | Sheep | 2008 | 3 | 16 / S | 16 / S | ≤ 4 / S | ≤ 1 / S | ≤ 1 / S | 2 / S | 2 / S | 1 / S | ≤ 2 / S | ≤ 1 / S | ≤ 1 / S | ≤ 0,25 / S | ≤ 0,5 / S |
| **Isolate** | **Species** | **Source** | **Year** | **Serotype** | **TIC** | **TCC** | **TZP** | **CAZ** | **FEP** | **ATM** | **IPM** | **MEM** | **AN** | **GM** | **TM** | **CIP** | **CS** |
| LiA24/2008 | Farm | Sheep | 2008 | 4 | 16/ S | 16/ S | ≤ 4 / S | ≤ 1 / S | ≤ 1 / S | 2 / S | 2 / S | 1 / S | ≤ 2 / S | ≤ 1 / S | ≤ 1 / S | ≤ 0,25 / S | ≤ 0,5 / S |
| LiA87/2009 | Farm | Cow | 2009 | NT | 32 / S | 32 / S | 8 / S | 2 / S | 2 / S | 8 / S | 1 / S | 0,5 / S | ≤ 2 / S | ≤ 1 / S | ≤ 1 / S | ≤ 0,25 / S | ≤ 0,5 / S |
| LiA144/2010 | Farm | Cow | 2010 | 10 | 32 / S | 64 / S | 8 / S | 4 / S | 2 / S | 16 / I | 2 / S | ≤ 0,25 / S | ≤ 2 / S | ≤ 1 / S | ≤ 1 / S | ≤ 0,25 / S | ≤ 0,5 / S |
| LiA87/2010 | Farm | Cow | 2010 | 4 | 32 / S | 32 / S | 8 / S | 4 / S | 2 / S | 4 / S | 2 / S | ≤ 0,25 / S | ≤ 2 / S | ≤ 1 / S | ≤ 1 / S | 0,5 / S | ≤ 0,5 / S |
| LiA91/2010 | Farm | Horse | 2010 | PA | 32 / S | 32 / S | 8 / S | 4 / S | 2 / S | 8 / S | 1 / S | ≤ 0,25 / S | ≤ 2 / S | ≤ 1 / S | ≤ 1 / S | ≤ 0,25 / S | ≤ 0,5 / S |
| LiA18/2011 | Farm | Horse | 2011 | 15 | 64 / S | 64 / S | 32 / I | 4 / S | 4 / S | 32 / R | ≤ 0,25 / S | ≤ 0,25 / S | 4 / S | 4 / S | ≤ 1 / S | ≤ 0,25 / S | ≤ 0,5 / S |
| LiA152/2012 | Farm | Pig | 2012 | 6 | 32 / S | 16 / S | 8 / S | 2 / S | ≤ 1 / S | 4 / S | 2 / S | 1 / S | ≤ 2 / S | ≤ 1 / S | ≤ 1 / S | ≤ 0,25 / S | ≤ 0,5 / S |
| LiA133/2003 | Zoo | Seal | 2003 | 9 | 32 / S | 32 / S | 8 / S | 4 / S | 2 / S | 16 / I | 2 / S | ≤ 0,25 / S | ≤ 2 / S | ≤ 1 / S | ≤ 1 / S | ≤ 0,25 / S | ≤ 0,5 / S |
| LiA91/2004 | Zoo | Dolphin | 2004 | 11 | 32 / S | 32 / S | 8 / S | 4 / S | 2 / S | 8 / S | 1 / S | 0,5 / S | ≤ 2 / S | ≤ 1 / S | ≤ 1 / S | ≤ 0,25 / S | 1 / S |
| LiA96/2004 | Zoo | Dolphin | 2004 | 11 | 32 / S | 32 / S | 8 / S | 4 / S | 2 / S | 8 / S | 2 / S | 0,5 / S | ≤ 2 / S | ≤ 1 / S | ≤ 1 / S | 1 / S | ≤ 0,5 / S |
| LiA63/2006 | Zoo | Kangaroo | 2006 | 1 | 64 / S | 64 / S | 16 / S | 8 / S | 4 / S | 32 / R | 2 / S | 0,5 / S | ≤ 2 / S | ≤ 1 / S | ≤ 1 / S | ≤ 0,25 / S | ≤ 0,5 / S |
| LiA192/2008 | Zoo | Kangaroo | 2008 | 9 | 64 / S | 64 / S | 8 / S | 4 / S | 2 / S | 16 / I | 2 / S | ≤ 0,25 / S | 4 / S | 2 / S | ≤ 1 / S | ≤ 0,25 / S | ≤ 0,5 / S |
| LiA198/2008 | Zoo | Saguim | 2008 | 6 | 32 / S | 32 / S | 8 / S | 4 / S | 2 / S | 8 / S | 1 / S | ≤ 0,25 / S | ≤ 2 / S | ≤ 1 / S | ≤ 1 / S | ≤ 0,25 / S | ≤ 0,5 / S |
| LiA134/2009 | Zoo | Saguinus imperator | 2009 | 6 | 32 / S | 64 / S | 8 / S | 4 / S | 2 / S | 16 / I | 1 / S | ≤ 0,25 / S | ≤ 2 / S | 3 / S | ≤ 1 / S | ≤ 0,25 / S | ≤ 0,5 / S |
| T2/12 | wild | Sea Turtle | 2010 | 6 | 16 / S | 16 / S | 8 / S | ≤ 1 / S | ≤ 1 / S | 2 / S | 1 / S | ≤ 0,25 / S | ≤ 2 / S | 2 / S | ≤ 1 / S | ≤ 0,25 / S | ≤ 0,5 / S |

Antimicrobials used for MDR and XDR classification are in pink. In green are MDR strains and yellow XDR strains.

Ticarcillin (TIC), ticarcillin+clavulanic acid (TCC), piperacillin+ tazobactam (TZP), ceftazidime (CAZ), cefepime (FEP), aztreonam (ATM), imipenem (IPM), meropenem (MEM), amikacin (AN), gentamicin (GM), tobramycin (TM), ciprofloxacin (CIP), and colistin (CS).
